# Viruses Infecting Cuban Honey Bees and Evolution of Deformed-Wing-Virus Variants

**DOI:** 10.3390/v18010148

**Published:** 2026-01-22

**Authors:** Poppy J. Hesketh-Best, Anais R. Luis, Declan C. Schroeder, Stephen J. Martin

**Affiliations:** 1 Department of Veterinary Population Medicine, College of Veterinary Medicine, University of Minnesota, St. Paul, MN 55108, USA; phesketh@igtp.cat (P.J.H.-B.); dcschroe@umn.edu (D.C.S.); 2 Centro de Investigaciones Apícolas, La Habana, Cuba; 3 School of Science, Engineering and Environment, University of Salford, Manchester M5 4WT, UK

**Keywords:** DWV, honey bee, Cuba, recombination

## Abstract

Cuba is in a unique situation in which it has a large (220,000 managed colonies) and isolated honey bee population due to a 60+ year ban on the importation of bees. Despite this, the ectoparasitic mite *Varroa destructor* arrived in 1996, and with it came deformed wing virus (DWV). In 2018, an island-wide survey detected varroa and DWV in 91% of colonies. In this study, we conducted a full-virome analysis on some of these samples, along with additional samples collected in 2021. For the first time, we detected two variants of Lake Sinai Virus and confirmed the absence of the normally widespread black queen cell virus in Cuba. We also detected both DWV-A and DWV-B master variants, with DWV-B being the dominant variant. Interestingly, the DWV-B/A recombinant was also detected, indicating that despite Cuba’s isolated nature, the pattern of DWV evolution mirrors that found in the USA and Europe. However, this pattern is not found in neighboring Latin America, China, or Japan, where the DWV-A master variant continues to be dominant. How and why two distinct evolutionary DWV pathways have arisen remain a mystery.

## 1. Introduction

Cuba, the largest Caribbean Island, is currently home to over 220,000 managed *Apis mellifera* (Apidae, Hymenoptera) honey bee colonies, in addition to a large free-living population [1]. An over-60-year ban on the importation of honey bees into Cuba has prevented the arrival of Africanized bees from neighboring countries. Studies using allozyme markers [2] and mitochondrial haplotypes (microsats) [3] have confirmed that the Cuban honey bee population is European (M and C lineages). The microsatellite data also showed that there is a homogeneous population of managed honey bees across the country without any regional differences, confirming the isolated nature of the population.

Despite the ban, in 1996, the ectoparasitic varroa mite, *Varroa destructor* (Varroidae, Mesostigmata), was first detected in the Matanzas province of Western Cuba and subsequently spread across the entire island. After initial losses of both managed and free-living colonies, natural varroa resistance arose, so no mite control methods have been used over the past 20+ years. This makes the Cuban population the largest varroa-resistant European honey bee population in the world [1]. Honey bees in Cuba [1], like those in many other countries [4], reduce the number of varroa mites by increasing their ability to detect and remove mite-infested worker cells [5]. This reduces the viral load within the colony due to there being fewer viral vectors (mites) [6].

An island-wide survey conducted in 2018 detected deformed wing virus (DWV, *Iflavirus aladeformis*, Iflaviridae, Picornavirales) in 91% of the analyzed colonies and 100% of the apiaries [7]. DWV has transformed from a minor honey bee pathogen to the most widespread and intensively studied insect pathogen in the world [8]. The dramatic rise in DWV’s prevalence is solely due to its association with the varroa mite. The mite introduced a new viral transmission route, i.e., inoculation directly into the hemolymph during mite feeding rather than the natural DWV transmission via food and mating. The lethal association between varroa and DWV has caused the death of millions of honey bee colonies and changed the viral landscape pertaining to honey bees. DWV, when transmitted by varroa, reduces the life expectancy of the infested honey bee pupae and leads to colony death when sufficient mite numbers are present. DWV is a multi-strain virus comprising four master variants, the common DWV-A and DWV-B variants, the rare DWV-C variant [8], and the potentially extinct DWV-D variant [9]. The master variant DWV-D was isolated from a dead pre-varroa colony from Egypt in 1977, and despite extensive searches of several hundred RNA-sequencing libraries, it now appears to have become extinct [9]. Its closest-related master variant is DWV-C, which has only been detected occasionally in viral surveys of varroa-infested honey bee colonies [10]. It is more common in stingless bees [11], small hive beetles [12], and, interestingly, in two varroa-free island populations of honey bees in the Azores [13]. In varroa-infested honey bees, the two major master variants, DWV-A and DWV-B, are almost always detected in honey bee surveys [8,14]. DWV-A was the first master variant to be detected in varroa-infested colonies [15]. However, longitudinal studies [10,16,17,18] have shown that the DWV-B master strain initially replaced DWV-A. During this process, various DWV-B/A recombinants emerged, which now appear to be dominant in both the US [19] and Europe [20]. In Latin America [11,18], China [21], and Japan [22], the DWV-A master variant remains dominant.

Here, we provide the first report of which DWV master variants are present in the isolated Cuban honey bee population.

## 2. Materials and Methods

Twelve samples, each containing around 50 adult workers, were collected from across Cuba in 2018 [7] and supplemented by new samples collected 2021 and stored in a freezer before being prepared for Oxford Nanopore Technologies (ONT) sequencing, as described in [19]. Briefly, DNA libraries were generated using Template-Switching RT Enzyme Mix (New England Biolabs, Ipswich, MA, USA) with an N6 TS–modified random primer (Thermo Fisher Scientific, Waltham, MA, USA) for first-strand synthesis and PrimeSTAR GXL polymerase (Takara Bio, San Jose, CA, USA) for second-strand synthesis [23,24]. Clean cDNA was then used to prepare Oxford Nanopore Technologies (ONT) sequencing libraries following the Native Barcoding Sequencing Kit (SQK-NBD114-96) protocol, with FPPE DNA Repair omitted. Pooled libraries were sequenced for 24 h on an R10 flow cell using a GridION (Oxford Nanopore Technologies, New York, NY, USA).

Reads were base-called and demultiplexed with Guppy v6.4 (high-accuracy model). Nanopore barcodes and adapters were trimmed using PoreChop v0.2.4. [25]. Trimmed reads were corrected and assembled with Canu v2.2 [26] using the following flags: nanapore maxInputCoverage = 2000 corOutCoverage = all corMinCoverage = 10 corMhapSensitivity = high minoverlap = 50 minread = 200 genomesize = 5000. Contigs ≥ 2 kb were manually binned in the anvi’o v7.1 interactive interface [27]. Anvi’o was used to profile contigs using Prodigal v2.6.3 [28]. Reads were mapped with Minimap2 v2.24 [29], and BAM files were generated with SAMtools v1.19.2 [30]. Coverage profiles were merged into a single database. Additional functional and taxonomic information was added using HMMER v3.4 against VOGs (https://vogdb.org/, accessed 31 December 2024), along with standard anvi’o HMMs, NCBI COGs, and KEGG Kofams [31,32]. Gene-level taxonomy was assigned using Kaiju v2.9 against the NCBI nr and RVDB databases (accessed 31 December 2023) [33]. Manual binning was guided by sequence composition (anvi’o dendrogram) and VOG HMMER hits.

## 3. Results

**Figure 1:**
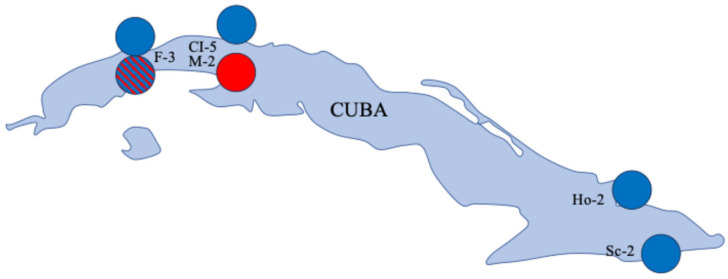
The locations and DWV variants (DWV-A, red; DWV-B, blue; and B-A recombinant, blue and red stripes) detected in Cuba. Locations: Funes (F-3), La Habana (CI-5), Managua (M-2), Holguin (Ho-2), and Santiago de Cuba (Sc-2). Within the single Funes pooled sample, contigs matching both the DWV-B and DWV-B-A recombinant were detected.

**Figure 2:**
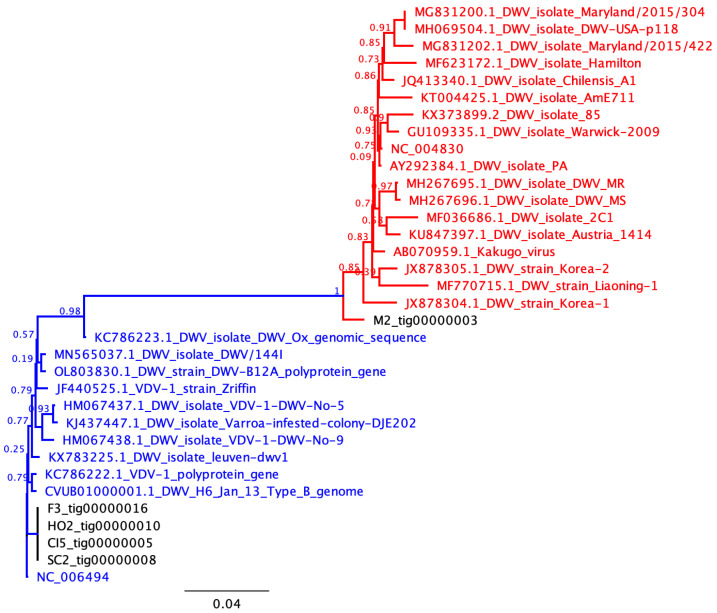
A phylogenic tree based on the VP2 region comparing the Cuban sample contigs, in black, with other samples, indicating the two main branches (DWV-A, in red, and DWV-B, in blue). The bar represents 4 substitutions per 100 nucleic acids. The nodes indicate FAstTree support values.

**Figure 3:**
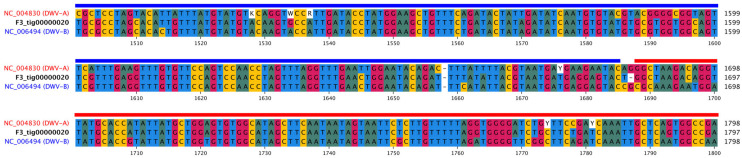
Multiple-sequence alignment of the putative recombinant genome with DWV-A (red, NC_004930) and DWV-B reference sequences (blue, NC_006494) indicated a possible recombination site within the GP1 region, located approximately 1678–1688 bp from the 3’ end of the reference genomes. Colored bar above the MSA indicates the structure of the putative recombinant genome (F3_tig00000020).

Due to RNA degradation caused by regular power cuts to the freezers in Cuba, we were only able to assemble eight (contigs) from the high-quality sequences obtained from the five samples, but these were located across Cuba (Table 1 and Figure 1), with contig lengths of between 2036 and 8448 (Table 1). Overall, the genomes obtained were fragmented, likely due to RNA degradation, limiting the completeness of the assemblies. We successfully sequenced a near-complete DWV-A genome in one sample (M2), DWV-B in four samples, and a DWV-B/A recombinant co-infection with DWV-B in one sample (Table 1, Figure 1 and Figure 2). The putative breakpoint for the recombinant genome occurred within the GP1 region (Figure 3). In addition, in one sample (CI-5, Table 1), we also detected two variants of lake Sinai virus (LSV, Sinhaliviridae, Nodamuvirales) for the first time in Cuba. No black queen cell virus (Dicistroviridae, Cripavirus) was detected in any of the samples.

## 4. Discussion

In this study, we did not detect black queen cell virus, which was detected in a previous viral survey of Cuba [7]. This is unusual since this virus is one of the most globally detected viruses in honey bee surveys [34,35]. Its absence may again reflect the isolated nature of the Cuban honey bee population. For the first time, we detected LSV in Cuba, which, unlike DWV, appears not to be an emerging disease but rather a highly variable multi-strain virus that has a stable association with *A. mellifera* [36]. The dominance of the DWV-B variant and the presence of the DWV-B/A recombinant in Cuban honey bees mirror the situation that was previously observed in Europe [20,37], the USA [19], and many other countries [14], with the DWV-A variant being replaced by DWV-B. The recombinant DWV-B/A sample detected in Cuba had DWV-B structural genes (VP1-VP4), meaning that previous RT-qPCR assays, e.g., [11], targeting the RdRP gene incorrectly indicated the presence of DWV-A rather than DWV-B/A recombinants. Interestingly, in all countries except Japan [22], where DWV-A is currently dominant, DWV-B has been detected but at very low prevalences, such as in Brazil (11%, n = 26) [11], Mexico (0.7–2%, n = 364) [18], and China (0.8%, n = 117) [38]. Although this study is based on only five samples, DWV-B was detected in 75% of the samples, with similar high prevalences of DWV-B detected in the UK (100%, n = 249) [10], US (56%, n = 217) [10], and Europe (82%, n = 50) [20]. This provokes us to ask why and how DWV-A has remained dominant over many decades in some countries and why it has been replaced by DWV-B, a development followed by the rise in DWV-B/A recombinants in other regions such as the USA and Europe. One proposed explanation is that superinfection exclusion by DWV-A may block DWV-B via the process of inter-genotype recombination meltdown [18], but this idea needs to be empirically tested.

## Figures and Tables

**Table 1:** Location, dates, contig lengths, and types of viruses from the various locations across Cuba that were successfully amplified.

Name	Date	Map Location	Contig	Virus	Contig Length	Accession Number
Managua	Dec-2021	M-2	tig00000003	DWV-A	8448	PX864649
Funes	Dec-2021	F-3	tig00000020	DWV-B/A recombinant	2053	PX864651
Funes	Dec-2021	F-3	tig00000016	DWV-B	2036	PX864650
La Habana	Dec-2021	CI-5	tig00000005	DWV-B	3616	PX864653
Santiago de Cuba	2018	Sc-2	tig00000008	DWV-B	5674	PX864654
Holguin	2018	Ho-2	tig00000010	DWV-B	3158	PX864652
La Habana	Dec-2021	CI-5	tig00000009	lake Sinai virus 3	2720	PX895024
La Habana	Dec-2021	CI-5	tig00000001	lake Sinai virus	5683	PX895023

## Data Availability

All data is either in the paper or deposited in GenBank.
